# Racial and Ethnic Differences in COVID-19 Vaccination Coverage Among Children and Adolescents Aged 5–17 Years and Parental Intent to Vaccinate Their Children — National Immunization Survey–Child COVID Module, United States, December 2020–September 2022

**DOI:** 10.15585/mmwr.mm7201a1

**Published:** 2023-01-06

**Authors:** Madeleine R. Valier, Laurie D. Elam-Evans, Yi Mu, Tammy A. Santibanez, David Yankey, Tianyi Zhou, Cassandra Pingali, James A. Singleton

**Affiliations:** ^1^Immunization Services Division, National Center for Immunization and Respiratory Diseases, CDC; ^2^Oak Ridge Institute for Science and Education, Oak Ridge, Tennessee; ^3^Leidos Inc., Atlanta, Georgia.

Some racial and ethnic groups are at increased risk for COVID-19 and associated hospitalization and death because of systemic and structural inequities contributing to higher prevalences of high-risk conditions and increased exposure (*1*). Vaccination is the most effective prevention intervention against COVID-19–related morbidity and mortality*; ensuring more equitable vaccine access is a public health priority. Differences in adult COVID-19 vaccination coverage by race and ethnicity have been previously reported (*2*,*3*), but similar information for children and adolescents is limited (*4*,*5*). CDC analyzed data from the National Immunization Survey–Child COVID Module (NIS-CCM) to describe racial and ethnic differences in vaccination status, parental intent to vaccinate their child, and behavioral and social drivers of vaccination among children and adolescents aged 5–17 years. By August 31, 2022, approximately one third (33.2%) of children aged 5–11 years, more than one half (59.0%) of children and adolescents aged 12–15 years, and more than two thirds (68.6%) of adolescents aged 16–17 years had received ≥1 COVID-19 vaccine dose. Vaccination coverage was highest among non-Hispanic Asian (Asian) children and adolescents, ranging from 63.4% among those aged 5–11 years to 91.8% among those aged 16–17 years. Coverage was next highest among Hispanic or Latino (Hispanic) children and adolescents (34.5%–77.3%). Coverage was similar for non-Hispanic Black or African American (Black), non-Hispanic White (White), and non-Hispanic other race^†^ or multiple race (other/multiple race) children and adolescents aged 12–15 and 16–17 years. Among children aged 5–11 years, coverage among Black children was lower than that among Hispanic, Asian, and other/multiple race children. Enhanced public health efforts are needed to increase COVID-19 vaccination coverage for all children and adolescents. To address disparities in child and adolescent COVID-19 vaccination coverage, vaccination providers and trusted messengers should provide culturally relevant information and vaccine recommendations and build a higher level of trust among those groups with lower coverage.

NIS-CCM is a nationally representative random-digit–dialed mobile telephone survey of households with children and adolescents aged 6 months–17 years. NIS-CCM interview data collected from 94,838 respondents during September 26, 2021–September 30, 2022,^§^ were used to assess racial and ethnic differences in COVID-19 vaccination coverage, parental intent to vaccinate, and behavioral and social drivers of vaccination among children and adolescents aged 5–17 years. Survey respondents were those who reported being knowledgeable about the child’s or adolescent’s vaccination status (parent). Interviews with parents of an unvaccinated child or adolescent, or a child or adolescent who received ≥1 COVID-19 vaccine dose^¶^ during December 2020–September 2022 were included. White persons were designated as the referent group for most racial and ethnic comparisons because this group has the largest population size and the most social advantage (*6*).

Kaplan-Meier estimation methods were used to calculate cumulative ≥1-dose COVID-19 vaccination coverage as of August 31, 2022.** First-dose vaccination month and year were hot deck imputed^††^ for 20.2% of children and adolescents with parent-reported vaccination but without a vaccination date (*7*). Differences in ≥1-dose coverage were assessed by race and ethnicity and stratified by sociodemographic subgroups. Pairwise comparisons were conducted to assess differences in ≥1-dose estimates for all races and ethnicities. Survey data during July 1–September 30, 2022,^§§^ (26,961) were pooled to calculate proportions of children and adolescents who 1) were unvaccinated, but parental intent to vaccinate was ascertained, 2) initiated COVID-19 vaccination (received ≥1 dose), 3) completed the primary series (received ≥2 doses), 4) received a monovalent booster (≥3 doses),^¶¶^ or 5) had an assessment of behavioral and social drivers of vaccination.*** For all analyses, p-values <0.05 were considered statistically significant. Analyses were performed using SAS (version 9.4; SAS Institute) and SAS-callable SUDAAN (version 11.0.3; Research Triangle Institute). Survey weights were used to adjust to the noninstitutionalized U.S. population of children and adolescents and to calibrate to CDC vaccine administration data. The cumulative Council of American Survey Research Organizations (CASRO) response rate^†††^ was 18.1%. This activity was reviewed by CDC and was conducted consistent with applicable federal law and CDC policy.^§§§^

By August 31, 2022, 33.2%, 59.0%, and 68.6% of persons aged 5–11, 12–15, and 16–17 years, respectively, had received ≥1 COVID-19 vaccine dose (Table 1). Coverage among Asian persons was higher than that among persons of other races and ethnicities, ranging from 63.4% (aged 5–11 years) to 91.8% (16–17 years), followed by Hispanic persons, with coverage ranging from 34.5% to 77.3% (Figure). Coverage was similar for Black, White, and other/multiple race persons aged 12–15 and 16–17 years. Coverage in Black children aged 5–11 years was 4.0 to 33.6 percentage points lower than that among Asian, Hispanic, and other/multiple race children of the same age (Supplementary Table 1, https://stacks.cdc.gov/view/cdc/122810).

**TABLE 1:** COVID–19 vaccination coverage with ≥1 dose as of August 31, 2022, among children and adolescents aged 5–17 years, by race and ethnicity* and demographic characteristics — National Immunization Survey–Child COVID Module, United States, September 26, 2021–September 30, 2022

Characteristic	Respondent distribution			Vaccinated,^†^ weighted % (95% CI)					
	Unweighted no.		Weighted %^§^ (95% CI)	Total (N = 94,838)	Asian (n = 4,962)	Black or African American (n = 10,103)	Hispanic or Latino (n = 19,805)	White (Ref)^¶^ (n = 51,606)	Other or multiple race (n = 8,362)
**Total**	**94,838**	**100.0**		**47.1 (46.5–47.7)**	75.2 (72.3–78.0)**	43.1 (41.4–44.8)**	49.1 (47.7–50.5)**	**45.0 (44.2–45.8)**	48.3 (46.0–50.6)**
**Age group, yrs**									
5–11 (Ref)	59,502	52.9 (52.4–53.5)		**33.2 (32.5–33.9)**	63.4 (59.3–67.5)**	29.8 (28.0–31.7)	34.5 (32.9–36.2)**	31.2 (30.3–32.0)	33.8 (31.2–36.6)
12–15	26,652	31.4 (30.9–32.0)		**59.0 (57.9–60.2)^††^**	89.2 (85.3–92.5)**^,††^	56.6 (53.3–60.0)^††^	63.9 (61.3–66.5)**^,††^	55.5 (54.0–57.1)^††^	58.6 (54.4–62.8)^††^
16–17	8,684	15.6 (15.2–16.1)		**68.6 (66.8–70.5)^††^**	91.8 (84.4–96.6)**^,††^	65.5 (60.0–71.0)^††^	77.3 (72.8–81.5)**^,††^	64.6 (62.3–66.9)^††^	67.8 (60.8–74.7)^††^
**Sex**									
Female	45,380	48.9 (48.3–49.4)		**48.1 (47.2–49.0)^††^**	75.9 (71.9–79.8)**	42.9 (40.6–45.3)**	49.9 (47.9–52.0)**	46.5 (45.3–47.6)^††^	47.6 (44.3–51.0)
Male (Ref)	48,974	51.1 (50.6–51.7)		**46.1 (45.2–46.9)**	74.3 (70.2–78.3)**	43.1 (40.8–45.5)	48.2 (46.2–50.1)**	43.7 (42.6–44.7)	48.7 (45.5–52.0)**
**Mother's highest education level**									
College degree or higher (Ref)	48,451	37.2 (36.6–37.7)		**63.6 (62.7–64.6)**	83.4 (80.3–86.3)**	55.4 (52.3–58.5)**	66.2 (63.6–68.7)**	62.0 (60.9–63.2)	70.6 (67.0–74.2)**
Some college	22,949	30.9 (30.3–31.4)		**38.8 (37.8–39.9)^††^**	66.2 (58.1–74.2)**^,††^	40.4 (37.7–43.3)**^,††^	44.2 (41.8–46.7)**^,††^	34.8 (33.4–36.2)^††^	40.1 (36.6–43.9)**^,††^
High school or equivalent	15,714	20.5 (20.0–21.0)		**35.5 (34.2–36.9)^††^**	68.9 (59.9–77.5)**^,††^	37.1 (33.7–40.7)**^,††^	43.0 (40.3–45.8)**^,††^	28.3 (26.7–30.0)^††^	34.2 (29.5–39.4)**^,††^
Less than high school	4,958	11.4 (11.0–11.9)		**39.0 (36.6–41.4)^††^**	NR^§§^	29.4 (24.4–35.2)^††^	46.6 (43.1–50.4)**^,††^	25.1 (22.2–28.3)^††^	36.5 (27.9–46.8)**^,††^
**Poverty status and household income**									
Above poverty, ≥$75,000 (Ref)	43,645	39.7 (39.1–40.2)		**55.8 (54.8–56.7)**	82.3 (77.6–86.4)**	53.4 (50.2–56.6)	56.9 (54.5–59.4)**	53.5 (52.4–54.7)	62.4 (58.8–66.0)**
Above poverty, <$75,000	21,269	24.9 (24.4–25.4)		**39.3 (38.1–40.5)^††^**	62.6 (55.3–69.9)**^,††^	39.8 (36.9–42.8)**^,††^	47.6 (44.9–50.4)**^,††^	32.8 (31.3–34.4)^††^	34.8 (30.9–39.0)^††^
Below poverty	8,965	13.2 (12.8–13.6)		**38.2 (36.3–40.1)^††^**	NR^§§^	34.0 (30.2–38.1)**^,††^	46.5 (43.1–50.0)**^,††^	28.0 (25.4–30.7)^††^	34.0 (28.1–40.8)^††^
Unknown income	20,959	22.2 (21.7–22.7)		**45.5 (44.2–46.8)^††^**	75.9 (71.1–80.4)**	41.8 (38.5–45.3)^††^	43.4 (40.5–46.3)^††^	44.2 (42.5–45.9)^††^	47.8 (42.9–52.9)^††^
**Health insurance**									
Other insurance (Ref)	65,506	63.4 (62.9–64.0)		**51.8 (51.0–52.6)**	79.6 (76.3–82.7)**	48.2 (45.8–50.6)	51.1 (49.2–53.1)	50.4 (49.5–51.3)	55.9 (52.9–59.0)**
Any Medicaid	21,601	32.1 (31.5–32.6)		**40.2 (39.1–41.4)^††^**	69.1 (62.0–76.0)**^,††^	38.4 (36.0–41.0)**^,††^	48.4 (46.1–50.8)**	32.5 (31.0–34.1)^††^	36.5 (32.8–40.4)^††^
Uninsured	3,458	4.5 (4.2–4.7)		**34.6 (31.3–38.1)^††^**	NR^§§^	34.8 (26.8–44.2)^††^	38.5 (32.9–44.6)**^,††^	25.3 (21.3–29.9)^††^	NR^§§^
**Urbanicity** ^¶¶^									
MSA, principal city (Ref)	29,633	32.6 (32.0–33.1)		**52.1 (51.0–53.3)**	74.2 (69.6–78.6)**	42.3 (39.9–44.9)**	52.7 (50.4–55.1)	53.7 (52.0–55.3)	53.3 (49.2–57.4)
MSA, nonprincipal city	45,475	53.5 (52.9–54.1)		**47.8 (47.0–48.7)^††^**	77.1 (73.4–80.7)**	43.8 (41.4–46.2)**	45.6 (43.7–47.7)^††^	47.6 (46.5–48.6)^††^	48.7 (45.5–52.0)
Non-MSA	14,688	13.9 (13.5–14.3)		**29.7 (28.4–31.1)^††^**	NR^§§^	43.4 (37.5–49.7)**	35.0 (31.0–39.3)**^,††^	26.3 (24.9–27.8)^††^	35.6 (31.0–40.8)**^,††^
U.S. Census Bureau region***									
Northeast (Ref)	18,269	15.2 (14.9–15.5)		**57.7 (56.3–59.1)**	82.4 (76.8–87.4)**	45.3 (41.4–49.3)**	53.4 (50.2–56.6)**	60.4 (58.6–62.2)	52.9 (47.5–58.6)**
Midwest	18,223	21.3 (20.9–21.6)		**40.4 (39.2–41.6)^††^**	66.3 (58.9–73.5)**^,††^	36.8 (33.1–40.9)^††^	40.7 (37.4–44.2)^††^	40.4 (39.0–41.9)^††^	37.1 (32.9–41.7)^††^
South	34,023	38.6 (38.2–39.1)		**42.3 (41.4–43.2)^††^**	71.2 (65.9–76.2)**^,††^	45.0 (42.9–47.2)**	45.6 (43.4–47.8)**^,††^	38.5 (37.3–39.7)^††^	40.4 (37.2–43.9)^††^
West	19,281	24.9 (24.4–25.4)		**52.2 (50.7–53.7)^††^**	76.8 (71.6–81.7)**	39.0 (32.9–45.7)**	49.7 (47.2–52.4)	50.9 (48.9–52.9)^††^	60.7 (56.2–65.2)**^,††^
**SVI of county of residence^†††^**									
Low (Ref)	31,027	28.0 (27.6–28.5)		**51.6 (50.6–52.7)**	77.6 (72.3–82.5)**	48.5 (44.6–52.6)	48.5 (45.6–51.4)	51.5 (50.2–52.7)	48.6 (44.8–52.6)
Moderate	31,119	37.3 (36.8–37.9)		**47.2 (46.1–48.2)^††^**	77.9 (73.5–82.0)**	44.9 (42.1–47.8)	48.5 (45.9–51.2)**	44.4 (43.1–45.7)^††^	50.7 (47.0–54.6)**
High	24,156	34.6 (34.1–35.2)		**44.1 (43.0–45.3)^††^**	70.3 (63.3–77.0)**	40.5 (38.0–43.1)^††^	49.0 (46.8–51.3)**	39.0 (37.4–40.6)^††^	46.1 (41.8–50.7)**
**Mask-wearing in indoor public spaces during previous 7 days**									
Always/Often wore mask (Ref)	49,336	51.0 (50.4–51.6)		**56.3 (55.3–57.3)**	77.4 (73.9–80.8)**	45.0 (43.0–47.1)**	54.8 (52.8–56.8)**	60.1 (58.6–61.6)	57.8 (54.5–61.1)
Sometimes/ Rarely/Never wore mask	44,475	49.0 (48.4–49.6)		**38.8 (38.0–39.6)^††^**	70.7 (65.5–75.7)**^,††^	38.4 (35.5–41.5)^††^	40.9 (38.8–43.0)**^,††^	37.1 (36.2–38.1)^††^	38.1 (35.0–41.4)^††^
**Influenza vaccination status since July 1, 2021^§§§^**									
≥1 dose influenza vaccine (Ref)	34,630	43.0 (42.3–43.7)		**63.4 (62.4–64.5)**	83.6 (79.6–87.2)**	53.9 (50.9–57.1)**	61.3 (58.9–63.6)**	64.6 (63.3–66.0)	65.8 (62.2–69.3)
Did not receive influenza vaccine	31,596	57.0 (56.3–57.7)		**32.2 (31.4–33.1)^††^**	55.3 (49.6–61.1)**^,††^	31.9 (29.7–34.3)^††^	36.6 (34.6–38.6)**^,††^	29.4 (28.3–30.5)^††^	31.2 (28.2–34.4)^††^
**Child/Adolescent ever had COVID–19**									
No (Ref)	61,551	63.4 (62.9–64.0)		**50.2 (49.4–51.0)**	75.8 (72.2–79.1)**	43.2 (41.2–45.2)**	51.2 (49.4–53.0)	49.4 (48.4–50.4)	52.2 (49.3–55.2)
Yes	32,310	36.6 (36.0–37.1)		**42.3 (41.3–43.3)^††^**	74.1 (68.8–79.0)**	42.9 (39.9–46.0)**	45.7 (43.4–48.0)**^,††^	38.9 (37.7–40.2)^††^	42.7 (39.0–46.5)^††^
**Mental health of child/adolescent**									
Excellent, very good, or good (Ref)	88,245	92.2 (91.9–92.5)		**46.6 (46.0–47.2)**	76.3 (73.4–79.1)**	42.8 (41.1–44.5)	48.4 (46.9–49.8)**	44.3 (43.5–45.1)	48.5 (46.1–50.9)**
Fair or poor	6,056	7.8 (7.5–8.1)		**53.3 (50.8–55.8)^††^**	NR^§§^	47.2 (40.5–54.5)	57.9 (51.1–64.9)^††^	53.9 (50.9–57.0)^††^	47.0 (39.8–54.8)

**Abbreviations:** MSA = metropolitan statistical area; NR = not reported; Ref = referent group; SVI = Social Vulnerability Index.

* Race and ethnicity were reported by the parent or guardian. Children and adolescents identified as Asian, Black or African American, White, or other or multiple races were reported by the parent or guardian as non-Hispanic. Children and adolescents identified as being of other or multiple races had more than one race category selected, or were identified as American Indian or Alaska Native, or Native Hawaiian or other Pacific Islander. Children and adolescents identified as Hispanic might be of any race.

^†^ Cumulative percentage vaccinated with ≥1 dose was estimated using Kaplan Meier survival analysis techniques with the event defined as the month and year of vaccination and the censoring variable defined as the date of interview.

^§^ Column percentages might not sum to 100 because of rounding.

^¶^ White persons were designated as the Ref for racial and ethnic comparisons because this group has the largest population size and the most social advantage. https://pubmed.ncbi.nlm.nih.gov/26599027/

** Statistically significant (p<0.05) difference compared with the indicated Ref (column) level (White).

^††^ Statistically significant (p<0.05) difference compared with the indicated Ref (row) level.

^§§^ Estimates were suppressed because they did not meet standards for data reliability (CI >20, relative SE >30, or sample size <30).

^¶¶^ Urbanicity was determined from household reported city and county of residence and was grouped into three categories: MSA principal city, MSA nonprincipal city, and non-MSA. MSA and MSA principal city were as defined by the U.S. Census Bureau (https://www.census.gov/programs-surveys/metro-micro.html). Non-MSA areas include urban populations not located within an MSA and completely rural areas.

*** https://www2.census.gov/geo/pdfs/maps-data/maps/reference/us_regdiv.pdf

^†††^ Categorization of National Immunization Survey–Child COVID Module data into an SVI level was based on zip code of residence reported by the respondent. https://www.atsdr.cdc.gov/placeandhealth/svi/index.html

^§§§^ Data on receipt of influenza vaccine were collected via interviews conducted during October 1, 2021–June 30, 2022.

**FIGURE:**
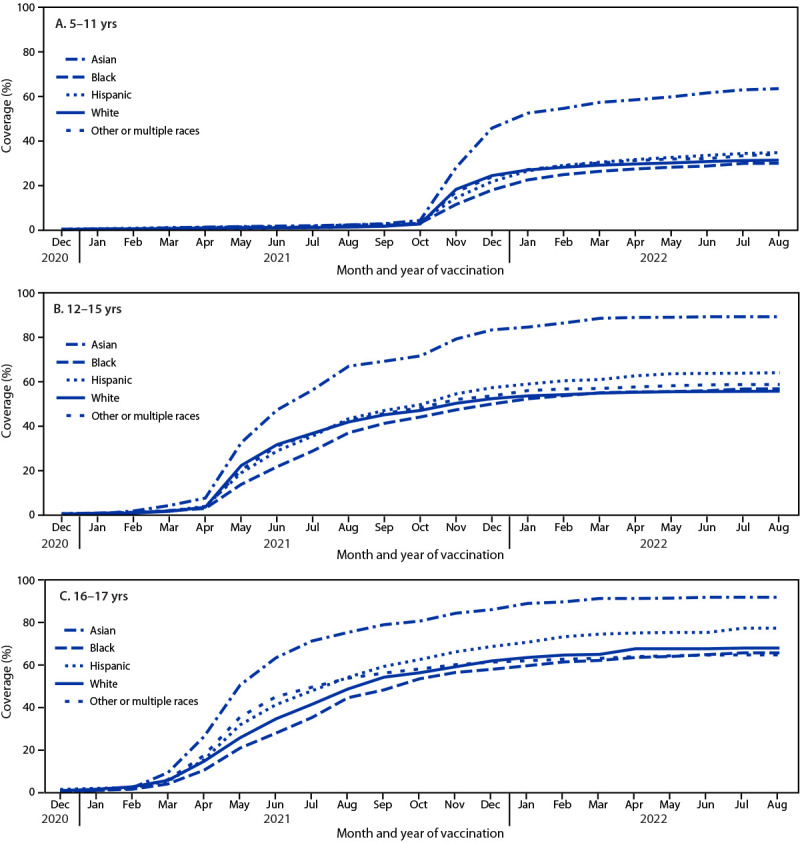
COVID-19 vaccination coverage estimates,* by race and ethnicity,^†^ among persons aged 5–11 years (A), 12–15 years (B), and 16–17 years (C) during December 2020–August 2022 — National Immunization Survey–Child COVID Module, United States, September 26, 2021–September 30, 2022 **Abbreviations:** Black = Black or African American; Hispanic = Hispanic or Latino. * ≥1 dose coverage; Kaplan-Meier survival analysis was used to estimate vaccination coverage based on the month and year of first dose receipt; estimates reflect the cumulative percentage vaccinated as of the end of each month. ^†^ Race and ethnicity were reported by the parent or guardian. Children and adolescents identified as Asian, Black, White, or other or multiple races were reported by the parent or guardian as non-Hispanic. Children and adolescents identified as being of other or multiple races had more than one race category selected or were identified as American Indian or Alaska Native, or Native Hawaiian or other Pacific Islander. Children and adolescents identified as Hispanic might be of any race.

Coverage with ≥1 COVID-19 vaccine dose by August 31, 2022, was higher among children and adolescents aged 12–17 years, those whose mothers had obtained a college degree, who lived in a household with yearly income ≥$75,000, who always or often wore a mask in public during the previous 7 days, and who had received ≥1 influenza vaccine dose (Table 1). Coverage among Asian and Hispanic children and adolescents was higher in most sociodemographic subgroups compared with coverage among White children and adolescents. Black, White, and other/multiple race children and adolescents had the largest variation in coverage by sociodemographic and behavioral characteristics.

Data collected during July 1–September 30, 2022, indicate that overall, 47.2% of children and adolescents received ≥1 COVID-19 vaccine dose; 43.3% completed the primary series (≥2 doses) (Table 2). Among children and adolescents aged 5–17 years, primary series coverage was highest among Asian persons. Monovalent booster dose coverage was low overall (14.7%) and was highest among Asian children and adolescents (22.4%) and lowest among Black children and adolescents (9.3%). Parents of White children and adolescents of all ages reported the highest level of reluctance^¶¶¶^ (40.3%) to have their child vaccinated.

**TABLE 2:** COVID-19 vaccination status among children and adolescents aged 5–17 years, and parental intent to vaccinate their children, by race and ethnicity* — National Immunization Survey–Child COVID Module, United States, July 1–September 30, 2022

Age group and race/ethnicity	Weighted % (95% CI)				
	Vaccinated, doses received			Unvaccinated, parents’ intent	
	Vaccinated ≥1 dose^†^	Primary series ≥2 doses	Booster, monovalent ≥3 doses	Definitely, probably, or unsure if will get child/adolescent vaccinated	Definitely or probably will not get child/adolescent vaccinated
**All, 5–17 yrs**	**47.2 (46.1–48.3)**	**43.3 (42.2–44.4)**	**14.7 (13.9–15.5)**	**19.5 (18.5–20.4)**	**33.4 (32.3–34.5)**
Asian	73.4 (68.4–77.8)^§^	69.3 (64.3–73.9)^§^	22.4 (18.3–27.0)^§^	14.9 (11.6–19.0)	11.7 (8.5–15.8)^§^
Black or African American	44.7 (41.6–47.8)	39.1 (36.2–42.1)	9.3 (7.8–11.1)^§^	28.9 (26.0–32.0)^§^	26.4 (23.5–29.5)^§^
Hispanic or Latino	49.0 (46.6–51.4)^§^	43.5 (41.2–45.9)	13.4 (11.9–15.0)^§^	26.2 (24.0–28.5)^§^	24.8 (22.7–27.1)^§^
White (Ref)^¶^	45.0 (43.5–46.5)	42.3 (40.9–43.8)	15.9 (14.9–17.0)	14.8 (13.6–15.9)	40.3 (38.7–41.8)
Other or multiple race	49.0 (44.8–53.3)	45.4 (41.2–49.7)	16.4 (13.3–20.1)	14.6 (12.1–17.5)	36.4 (32.3–40.7)
**5–11 yrs**	34.1 (33.0–35.3)	29.7 (28.6–30.8)	5.5 (5.0–6.0)	25.3 (24.1–26.6)	40.6 (39.2–41.9)
Asian	63.5 (57.3–69.2)^§^	57.1 (51.1–62.9)^§^	10.9 (8.0–14.7)^§^	20.2 (15.7–25.6)	16.3 (11.8–22.0)^§^
Black or African American	32.7 (29.6–35.9)	27.3 (24.4–30.3)	4.5 (3.4–5.8)^§^	36.3 (32.6–40.1)^§^	31.1 (27.5–34.8)^§^
Hispanic or Latino	35.2 (32.6–37.8)^§^	28.8 (26.5–31.2)	4.6 (3.7–5.7)^§^	33.3 (30.5–36.3)^§^	31.5 (28.7–34.4)^§^
White (Ref)^¶^	32.0 (30.5–33.5)	29.0 (27.5–30.4)	6.0 (5.3–6.7)	19.1 (17.5–20.7)	49.0 (47.1–50.8)
Other or multiple race	33.0 (28.8–37.4)	28.8 (24.9–33.1)	5.0 (3.7–6.7)	19.7 (16.3–23.7)	47.3 (42.3–52.4)
**12–15 yrs**	58.2 (56.0–60.5)	55.1 (52.8–57.3)	21.8 (20.1–23.5)	14.9 (13.2–16.7)	26.9 (24.8–29.1)
Asian	87.3 (78.6–92.8)^§^	86.6 (77.9–92.2)^§^	33.7 (24.6–44.3)^§^	8.2 (3.7–17.0)	4.5 (2.1–9.5)^§^
Black or African American	58.2 (51.4–64.7)	53.1 (46.5–59.6)	15.2 (11.6–19.6)^§^	20.0 (15.1–26.0)^§^	21.7 (16.2–28.5)^§^
Hispanic or Latino	61.8 (56.9–66.4)^§^	57.8 (52.9–62.5)	20.7 (17.5–24.3)	20.8 (16.9–25.3)^§^	17.5 (13.9–21.6)^§^
White (Ref)^¶^	54.5 (51.5–57.5)	52.2 (49.2–55.2)	22.4 (20.2–24.8)	12.0 (10.1–14.4)	33.5 (30.5–36.5)
Other or multiple race	62.0 (53.6–69.7)	57.5 (49.2–65.5)	26.1 (19.4–34.2)	10.0 (6.4–15.4)	28.0 (20.9–36.4)
**16–17 yrs**	68.8 (65.3–72.1)	65.6 (62.1–68.9)	31.3 (28.5–34.3)	9.0 (7.0–11.4)	22.2 (19.3–25.6)
Asian	87.3 (66.1–96.1)^§^	86.2 (65.9–95.3)^§^	46.7 (31.3–62.8)	6.2 (1.2–26.3)	6.4 (1.1–29.8)^§^
Black or African American	66.4 (55.5–75.8)	59.5 (48.9–69.2)	17.5 (11.9–25.2)^§^	16.7 (10.0–26.6)^§^	16.9 (9.8–27.6)^§^
Hispanic or Latino	75.0 (66.7–81.8)^§^	70.4 (62.1–77.6)	32.3 (26.0–39.3)	9.8 (5.6–16.4)	15.2 (9.8–23.0)^§^
White (Ref)^¶^	65.8 (61.1–70.1)	63.5 (58.9–67.9)	33.5 (29.7–37.5)	7.0 (4.8–10.1)	27.2 (23.1–31.7)
Other or multiple race	68.6 (55.8–79.0)	67.9 (55.2–78.3)	29.1 (19.5–41.1)	9.3 (4.4–18.7)	22.1 (13.1–34.9)

**Abbreviation:** Ref = referent group.

* Race and ethnicity were reported by the parent or guardian. Children and adolescents identified as Asian, Black or African American, White, or other or multiple races were reported by the parent or guardian as non-Hispanic. Children and adolescents identified as being of other or multiple races had more than one race category selected or were identified as American Indian or Alaska Native, or Native Hawaiian or other Pacific Islander. Children and adolescents identified as Hispanic might be of any race.

^†^ Proportions of children and adolescents who received ≥1 dose COVID-19 vaccine based on July–September 2022 interview data might not match Kaplan-Meier vaccination coverage estimates, which use more months of data (September 26, 2021–September 30, 2022) and a different analytic method.

^§^ Statistically significant (p<0.05) difference compared with White persons.

^¶^ White persons were designated as the Ref for racial and ethnic comparisons because this group has the largest population size and the most social advantage. https://pubmed.ncbi.nlm.nih.gov/26599027/

Parents of vaccinated children and adolescents reported high levels of confidence in the importance (93.1%) and safety (78.8%) of vaccination overall and by race and ethnicity (Supplementary Table 2, https://stacks.cdc.gov/view/cdc/122811). Confidence in vaccine importance remained high (76.7%), but confidence in vaccine safety was considerably lower (40.2%) among parents of unvaccinated children and adolescents who might get their child vaccinated (reachable children****). Parents of reluctant and reachable children and adolescents reported substantially lower percentages of friends and family had vaccinated their children and adolescents (reflection of social norms) (6.4%–21.2%) and reported having received a provider recommendation (27.9%–35.9%) for vaccination compared with parents of vaccinated children (72.9% and 61.8%, respectively). The magnitude of these differences was similar by race and ethnicity.

Overall, reluctant parents expressed less favorable attitudes and opinions regarding vaccination than did parents of vaccinated and unvaccinated but reachable children. Among all reluctant parents, those of Black and Hispanic children and adolescents reported the highest levels of concern about their child getting COVID-19 and expressed the most confidence in the importance of the vaccine.

## Discussion

COVID-19 vaccination coverage was low among children and adolescents aged 5–17 years overall, but highest among Asian and Hispanic children and adolescents. By August 31, 2022, Asian children and adolescents had substantially higher coverage than did all other children and adolescents overall and when stratified by factors associated with lower coverage for all children and adolescents, indicating a willingness across demographic and behavioral characteristics in this population to receive vaccination. During July 1–September 30, 2022, overall and among all racial and ethnic groups, most children and adolescents who initiated a primary COVID-19 vaccination series also completed the primary series, an encouraging sign of COVID-19 vaccine access and acceptance among parents who intend to vaccinate, but efforts are needed to achieve much higher coverage levels for all children and adolescents.

Lower coverage with ≥1 COVID-19 vaccine dose associated with some demographic and behavioral characteristics point to opportunities to improve coverage. Frequent mask use in public and receipt of influenza vaccine were associated with higher COVID-19 vaccination coverage among all children and adolescents; however, among Black and Hispanic children and adolescents with these characteristics, the increase in coverage was smaller. Less than one half of parents of Black and Hispanic children and adolescents had confidence in COVID-19 vaccine safety, which might indicate reluctance to be vaccinated among a population receptive to other public health behaviors. During July 1–September 30, 2022, large proportions of Hispanic and Black children and adolescents were unvaccinated but reachable (26% and 29%, respectively), suggesting that coverage might increase over time with strengthened public health interventions. A higher proportion of parents of other/multiple race and White children and adolescents were reluctant to vaccinate their child (36% and 40%, respectively) than were considered reachable (15%), suggesting potential difficulty achieving high vaccination coverage among these children and adolescents.

Implementation of evidence-based practices described in CDC’s COVID-19 Vaccination Field Guide^††††^ could help increase vaccine coverage. Community members should serve as trusted messengers to advocate for vaccination among parents of unvaccinated children and adolescents and should deliver tailored messages to strengthen confidence in vaccine safety and importance. Provider recommendation is an impactful driver of vaccination (*8*). A multifaceted approach with community collaboration and provider recommendation are essential to increasing childhood COVID-19 vaccination coverage.

The findings in this report are subject to at least five limitations. First, the survey response rate was low (18.1%). Data were weighted to account for household and provider nonresponse and for households without telephones and weighted to the COVID-19 vaccine administration data (≥1 doses) reported by jurisdictions to CDC. However, some bias might remain. Second, child and adolescent COVID-19 vaccination receipt was parent-reported and might be subject to recall or social desirability biases. However, limited recall bias is expected because of the recency of COVID-19 vaccination recommendations. Third, small sample sizes were available for American Indian or Alaska Native and for Native Hawaiian or other Pacific Islander children and adolescents; therefore, these data were aggregated in the other/multiple race category. Fourth, aggregated racial and ethnic data might obscure differences in coverage that are apparent in disaggregated subgroups (*9*). Data on racial and ethnic subgroups were collected, but the sample size was inadequate to analyze the disaggregated data. Finally, reporting of month and year of vaccination was incomplete for one fifth of children and adolescents who were reported to be vaccinated, requiring imputation of missing dates.

Public health efforts to increase coverage with the primary COVID-19 vaccination series in all age groups and the bivalent COVID-19 booster dose^§§§§^ among eligible persons should continue. These efforts should be tailored to differences in parental intent, behavioral and social drivers of vaccination, and by the child’s or adolescent’s age, race, and ethnicity. Programs should provide culturally relevant information and employ evidence-based strategies, including tailored messages delivered by trusted messengers and strong recommendations from vaccination providers, to increase vaccine confidence and coverage among all groups, and to eliminate the disparities for those with lower vaccination coverage.

**Summary**

**What is already known about this topic?**

Some racial and ethnic groups are at increased risk for COVID-19–associated morbidity and mortality because of systemic and structural inequities. Vaccination is effective in preventing severe COVID-19–related outcomes.

**What is added by this report?**

Among children and adolescents aged 5–17 years, ≥1-dose COVID-19 vaccination coverage was low overall, but highest among Asian and Hispanic or Latino children and adolescents. Parental intent to vaccinate their child varied by the child’s age, race, and ethnicity. Parents of unvaccinated children and adolescents reported low confidence in vaccine safety, and a low percentage reported receipt of a provider vaccination recommendation.

**What are the implications for public health practice?**

To increase overall coverage and address disparities in child and adolescent COVID-19 vaccination coverage, providers and trusted messengers should provide culturally relevant information and vaccine recommendations.

## Footnotes

* CDC’s Advisory Committee on Immunization Practices (ACIP) issued an interim recommendation for use of the Pfizer-BioNTech COVID-19 vaccine for persons aged ≥16 years on December 12, 2020, 12–15 years on May 12, 2021, and 5–11 years on November 2, 2021. ACIP recommended use of an additional homologous primary dose of Pfizer-BioNTech vaccine after an initial series in immunocompromised persons aged ≥12 years on August 13, 2021. ACIP recommended monovalent booster doses for persons aged 16–17 years on December 9, 2021, 12–15 years on January 5, 2022, and 5–11 years on May 19, 2022.

† Race and ethnicity of the child or adolescent were reported by the parent or guardian and were available for all study participants. Asian, Black, White, or other/multiple race children and adolescents were reported by the parent or guardian to be non-Hispanic. Other/multiple race children and adolescents had more than one race category selected, or were identified as American Indian or Alaska Native, or Native Hawaiian or other Pacific Islander. Hispanic children and adolescents might be of any race.

§ Respondents interviewed during September 26, 2021–September 30, 2022, were included; month and year of vaccination occurred during December 2020–September 2022.

¶ Assessed by response to the question, “Has [child’s name] received at least one dose of a COVID-19 vaccine?” Month and year of first dose was assessed by asking, “During what month and year did [child’s name] receive [his/her] first COVID-19 vaccine?”

** Kaplan-Meier estimation methods were used to calculate estimated cumulative vaccination coverage as of the end of each month from December 2020 through August 2022 using data from interviews conducted during September 26, 2021–September 30, 2022, in which the event was defined as the month and year of receipt of first dose of COVID-19 vaccine and was censored by date of interview. Vaccination status was assigned as of the end of the month before the interview.

†† Data were imputed using hot deck imputation (replacing missing values with observed values from a respondent with similar characteristics) from donor pools matched for month of interview, age group, region, and race and ethnicity.

§§ Interview data were restricted to the previous 3 months (July 1–September 30, 2022) to provide the most current assessment of vaccination coverage, parental intent, and behavioral and social drivers of vaccination. Estimates of vaccination coverage represent the cumulative percentage of children and adolescents vaccinated as of approximately the midpoint of this interview period (mid-August 2022), and estimates of coverage with ≥1 dose might differ from the Kaplan-Meier estimates as of August 31, 2022.

¶¶ Information on whether the child or adolescent was immunocompromised was not ascertained in NIS-CCM; thus, booster dose coverage estimates might include immunocompromised children and adolescents who received 3 primary COVID-19 vaccine doses.

*** Assessed by responses to six questions covering 1) concern about the child or adolescent getting COVID-19, 2) confidence in COVID-19 vaccine safety, 3) confidence in COVID-19 vaccine importance, 4) percentage of friends and family who had vaccinated their children (social norms), 5) health care provider recommendation for COVID-19 vaccination, and 6) school COVID-19 vaccination requirements. https://www.cdc.gov/vaccines/imz-managers/nis/downloads/NIS-CCM-Questionnaire-Q3-2022.pdf

††† The CASRO response rate is the product of three rates: 1) the proportion of telephone numbers that can be identified as either for business or residence (resolution rate), 2) the proportion of qualified households that complete the screening process (screening rate), and 3) the proportion of contacted eligible households for which a completed interview is obtained (cooperation rate).

§§§ 45 C.F.R. part 46.102(l)(2), 21 C.F.R. part 56; 42 U.S.C. Sect. 241(d); 5 U.S.C. Sect. 552a; 44 U.S.C. Sect. 3501 et seq.

¶¶¶ Unvaccinated children and adolescents whose parents responded that they definitely or probably will not vaccinate their child (reluctant).

**** Unvaccinated children and adolescents whose parents responded that they definitely, probably will, or are unsure if they will vaccinate their child (reachable).

††††
https://www.cdc.gov/vaccines/covid-19/downloads/vaccination-strategies.pdf

§§§§ As of December 9, 2022, persons aged ≥6 months are recommended to receive a bivalent booster dose, even if they already received a monovalent booster dose. https://www.cdc.gov/coronavirus/2019-ncov/vaccines/stay-up-to-date.html
